# Effect of Substitution of Hydrogen Atoms in the Molecules of Anthrone and Anthraquinone

**DOI:** 10.3390/molecules26020502

**Published:** 2021-01-19

**Authors:** Małgorzata Szymańska, Irena Majerz

**Affiliations:** Faculty of Pharmacy, Wroclaw Medical University, Borowska 211a, 50-556 Wroclaw, Poland; m.szymanska@umed.wroc.pl

**Keywords:** anthrone, anthraquinone, aromaticity, QTAIM

## Abstract

The geometry of anthrone and anthraquinone—natural substances of plant origin—was investigated under the substitution of hydrogen atoms in side aromatic ring and, for anthrone, also in the central ring. A significant influence of substitution on geometry expressed by the angle between the side rings was shown. The geometry changes are connected with the changes of electron density and aromaticity of the anthrone and anthraquinone rings. The flexibility of the investigated compounds was confirmed by comparison of the optimized molecules and the molecules in the crystal state where the packing forces can influence the molecular geometry.

## 1. Introduction

Anthrones are compounds of natural origin extracted from plants. The broad spectrum of biological and medical properties [1,2] means that interest in monoanthrones has not diminished over the years. Monoanthrones have antimicrobial [3], cytotoxic [4], anti-HIV [4], antifungal [5], antiviral [6], phototoxic [7,8], antioxidant [9,10], anticancer [11,12], and anti-inflammatory [13] properties. Anthraquinones have antifungal [1], cytotoxic [4], anti-HIV [4], antioxidant [1], antibacterial, antiviral, and antitumor [6,14] properties.

**Scheme 1:**
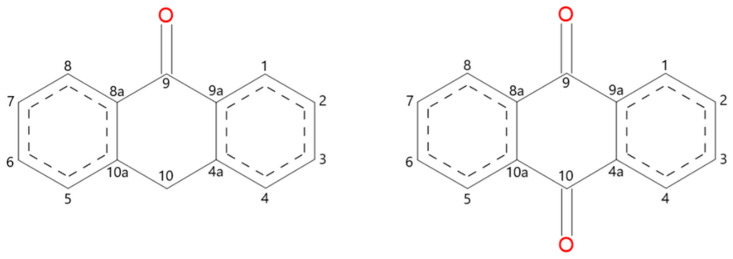
Molecular structure and atom numbering of anthrone and anthraquinone.

Anthrones and anthraquinones are tricyclic compounds (Scheme 1). Two side rings have an aromatic character, while the central ring is aliphatic. The aliphatic character of the central ring affects the non-planar structure of anthrones. It can be expected that the oxygen atom substituted to the central ring flattens the molecule.

In the literature, there is a lot of information linking the physical and pharmaceutical properties of anthrones and anthraquinones with substituents of the aliphatic and aromatic ring and molecular geometry. According to Gow-Chin Yen [10], there is a relationship between the presence of the ketone groups in the central ring and substitution of the lateral aromatic rings and the antioxidant properties of anthrones. The antioxidant with one ketone group at the central ring showed the strongest antioxidant activity. The antioxidant activity of numerous compounds results from the presence of the OH group. Moreover, unsubstituted anthraquinone showed the least antioxidant properties. Kamei [15], who studied the effect of anthraquinones on inhibiting cell growth, came to similar conclusions. It turned out that the presence of OH groups attached to lateral aromatic rings can have a significant impact on the antitumor properties of the tested compounds. The activity of anthrones is influenced not only by the number of the OH groups in the aromatic rings, but also by their location. According to Cai [16], ortho-dihydroxy substitution in the anthraquinone molecule significantly increases the scavenging effect. Marković [17] performed an analysis of the bond dissociation enthalpy (BDE) for all OH sites of emodin. In his opinion, a significant role in antioxidant properties is played by the OH group substituted to the third carbon atom [17] (Scheme 1).

The physicochemical properties and reactivity of the cyclohexa-2,5-dienone analogs with the heteroatom in the ring indicate a partially aromatic nature of the ring [18]. In the case of anthrone, the partially aromatic character of the central ring can be additionally influenced by the presence of side aromatic rings with mobile π electrons. It has been evidenced that the enol form present in anthrone stabilizes the aromaticity of the rings [19] and high π-electron delocalization in the condensed rings suggests that the central ring in anthrone and anthraquinone may change aromaticity under the influence of electron density change in the molecule caused by the presence of substituents.

For a better understanding of the therapeutic and physicochemical properties and mechanism of action of anthrones and anthraquinones, it is necessary to perform an analysis of the geometry of the single molecule and its electronic structure. In this work, we have undertaken a systematic theoretical study to analyse the structural parameters of anthrones and anthraquinones under substitution with electron donating and electron withdrawing groups. The electron donating the NH_2_ group characterized by the strongest donating properties (σ_p_ = −0.66) was chosen. (The substituent constant σ_p_ is a measure of the total polar effect exerted by substituent in para position. It is positive for electron withdrawing and negative for electron donating substituent.) Similarly, the electron withdrawing NO_2_ group has the strongest electron withdrawing properties (σ_p_ = 0.77) among the substituents. Because it can be expected that a substituent attached to the aromatic ring can affect the geometry of the investigated molecules, a systematic study of the structural parameters under substitution was performed. By single and multiple substitutions in the aromatic ring of each of these groups, a systematic study of the effect of donating or withdrawing the charge to the ring was carried out.

**Scheme 2:**
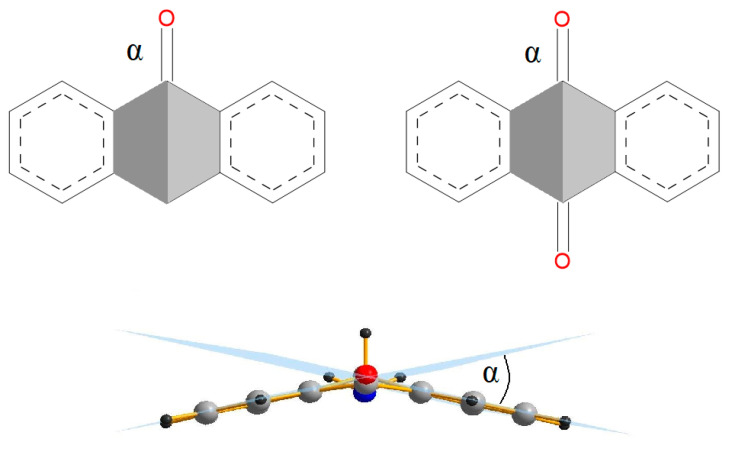
Definition of the α angle for monoanthrones and anthraquinones.

**Scheme 3:**
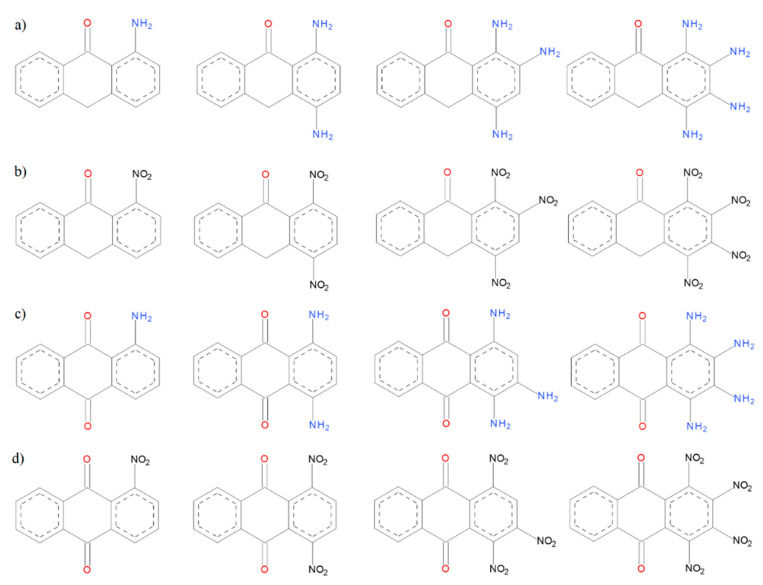
(**a**) Anthrone structures with the substituents with electron-donor properties, (**b**) anthrone structures with substituents with electron-withdrawing, (**c**) anthraquinone structures with substituents with electron-donor properties, and (**d**) anthraquinone structures with substituents with electron-withdrawing.

The main structural parameter for anthrones and anthraquinones is the alpha angle (Scheme 2) between two planes formed by four atoms of the middle ring. To investigate the influence of substitution on the geometry of the anthrone, the structures with NO_2_, CHO, COOH, CH_3_, CH_2_CH_3_, NH_2_, OH, Cl, and C(CH_3_)_3_ substituents in the central ring were also optimized. An additional group of the compounds used in the analysis are the structures delivered from the Cambridge Structural Database (CSD) [20], which were optimized and compared with the X-ray structures. The difference between the solid state structure and the optimal structure shows how much changes in the environment of a molecule can affect its geometry. The structures of anthrones and anthraquinones with electron-donor and electron-withdrawing substituents at the aromatic side ring were optimized (Scheme 3). The aliphatic character of the central ring of anthrones and anthraquinones determines the non-planarity of the molecules.

The presence of two ketone groups in the aliphatic ring causes its flattening, which, together with the aromatic CC bonds common with the aromatic side rings, can influence the aliphatic character of the central ring. It can be assumed that the flattening of the central ring is related to changes in the electron density. To characterize these changes, the QTAIM (quantum theory of atom in molecule) [21] method was used, which enables the description of the electron density both in the centre of the molecule ring and on the aromatic chemical bonds. Because it can be assumed that the electron density in the middle ring is sensitive to substitution, the electron density parameters for the central ring critical point were correlated with the α angle. As the changes in geometry are related to changes in the electron density, and these in turn are related to changes in aromaticity, for all rings in the investigated compounds, the aromaticity was characterized using the HOMA (*harmonic* oscillator model of aromaticity) [22].

### Computational Details

The investigated molecules were optimized using a Gaussian 16 package [23] at DFT-D3 B3LYP/6-311++G** level [24,25], which included Grimme dispersion [26]. To check that the resultant geometry reached the energy minimum, vibrational frequencies were calculated. The wave function evaluated for the optimized molecules was used as the input to the AIMALL program [21].

## 2. Results

### 2.1. Geometry of Investigated Compounds

**Scheme 4:**
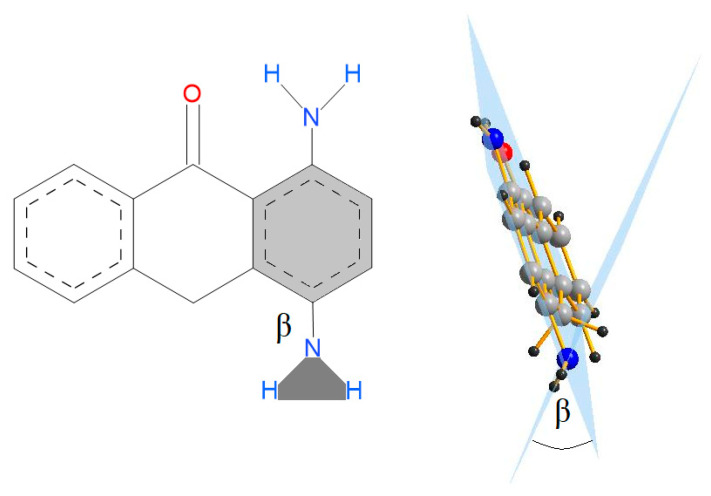
Definition of the β angle.

The main geometric parameter for the studied molecules—the angle α—is presented in Scheme 2 and collected in Table S1 (Supplementary Materials). The α angle is very sensitive to the substitution in the aliphatic and aromatic ring and changes from 0 to 41°. The values of the α angle for optimized anthrone and anthraquinone molecules as well as for the structures in the crystal are collected in Table 1. The α angle is more sensitive to substitution in the anthrone central ring and is affected not only by the character of the substituent, but also by its size and axial or equatorial orientation. Most of the computed structures have a substituent in the axial position. The exception is 10-amino-10*H*-anthracen-9-one, for which the structure with the axial substituent, as well as 10-*tert*-butyl-10H-anthracen-9-one, could not be obtained. For two 10-*t*-butyl-9,10-dihydro-9-anthracenone structures, the difference between the α angle for the axial and equatorial substituent location is 13.321°. In the 10-methyl-10*H*-anthracen-9-one structure taken from the CSD database [27], the slope angle for the methyl substituent and for the hydrogen atom in relation to the plane of the middle ring is very similar. Therefore, it cannot be clearly stated that it is a structure with a substituent in the axial or equatorial position. More limited changes of the α angle are observed for structures with substituents in the side ring. Minimal changes of the α angle were obtained for anthraquinone structures with substituents in the benzene ring. It is interesting to note the multiple substitution of the aromatic ring with electron-donor and electron-withdrawing substituents because of the steric hindrances between adjacent groups causes bending of the substituted ring. To better understand how substituents with electron-donor and electron-withdrawing properties are arranged against the ring plane, the β angle between the plane of the substituted ring and the substituent plane (Scheme 4) was determined (Table 2). Substitution of the amino group next to the ketone group causes formation of weak hydrogen bonds (H⋯O 1.87–1.89 Å), which reduces the β angle. Close location of the amino groups causes an increase of the β angle, which is associated with the steric hindrance. It is known that the nitro group tends to be located in the plane of the aromatic ring to which the group is substituted. Substitution to the anthrone side ring of a nitro group located next to the ketone group of the middle ring causes the nitro group to swing out the plane of the benzene ring and the β angle increases. The β angle decreases by about 30° when the nitro group is not close to the ketone or another nitro group. The nitro groups are larger than the amino groups; therefore, substitution of four nitro groups to the aromatic ring causes the ring to become non-planar and the β angles for the substituted groups to be larger than in the case of amino substitution. Another interesting feature of the structures substituted with many amino and nitro groups is elongation of the C–N bond linking the substituent with the aromatic ring. The close location of amino and carbonyl groups reduces the length of the C–N bond in both anthrones and anthraquinones. The bond is more elongated if the number of substituents increases and the elongation is more significant for amino groups substituted to anthrones when, for the substitution with the nitro group, the elongation of the C–N bond is more significant for anthraquinones.

It is characteristic that the carbonyl group substituted in the central ring of the anthrone causes significant flattening of the molecule compared with 9,10-dihydroanthracene, investigated previously, for which the α angle is 39.036° [33]. The aliphatic ring of anthraquinone is planar with an α angle of 0.003°. The α angle is very sensitive to substitution, especially in the anthrone central ring, but also to the substitution in the aromatic side ring. Changes of the α angle are more prominent for anthrone than for anthraquinone. Substitution of the side ring with an NH_2_ and NO_2_ group influences the α angle, and these changes are more significant for the NO_2_ group and for anthrone compared with anthraquinone. Multiple substitution of the aromatic ring is connected with twisting of the substituent group against the aromatic ring and elongation of the C–N bond length between the aromatic ring and the substituent. Comparison of the α angle for the optimized molecule and the same molecule in crystal (Table 1) confirms the flexibility of this angle, which can be changed as a result of packing in the crystal lattice. For 10-methyl-9,10-dihydro-9-anthracenone, the angle of 16.168° for the optimized molecule changes by up to 4.92°.

### 2.2. Electron Density at Central-Ring-Critical Point

In the frame of quantum theory of atoms in molecules (QTAIM), the molecule is treated as electron density, ρ(r), characterized by a system of critical points (CP) for which the gradient of the electron density vanishes. Diagonalization of the Hessian of electron density gives non-zero eigenvalues and their number and the sum of their signs describes a characteristic of the critical point. The maximum of ρ(r) represents the nucleus when the minimum of ρ(r) corresponds to the cage critical point. The bond critical point (BCP) and ring critical point (RCP) are the saddle points of the electron density. The gradient path of electron density linking the atoms located at its maximum is a chemical bond with a BCP at the minimum along the bond path and maximum along the directions perpendicular to the bond path [34]. From the BCP, two gradient paths extend to the atoms linked by the chemical bond. Except for the chemical bond, depending on the BCP parameters, the gradient path is also important for an interaction between two atoms [35,36]. The quantitative description of the molecule is connected with the analysis of the topological parameters of critical points [37].

Changes of the molecular geometry are usually reflected in the changes of electron density. Looking at the geometric changes of the investigated anthrone and anthraquinone molecules, it can be expected that the significant changes of electron density will be related to the central aliphatic ring, especially to the ring-critical point (RCP) of the aliphatic ring, and can be correlated with the α angle. The aromaticity of a ring may be related to its electron density. It has been shown that the parameters used in the QTAIM theory to describe the electron density, such as electron density and potential and kinetic energy at the critical point of the ring, can be used as parameters describing the aromaticity of the ring [38].

**Figure 1:**
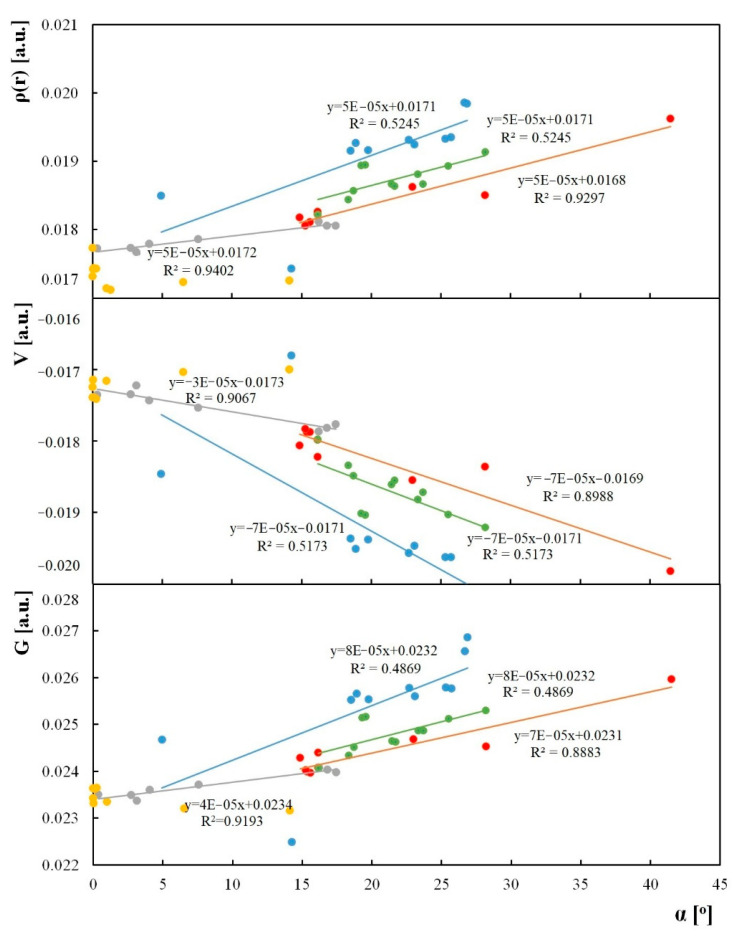
Correlations of quantum theory of atoms in molecules (QTAIM) parameters at the ring-critical point of the central ring. A-electron density, b-kinetic-energy density, c-potential-energy density, where blue-structures from the Cambridge Structural Database (CSD); red-anthrones with a substituent in the central ring, grey anthrones with substituent NO_2_ and NH_2_, yellow-anthraquinones with substituent NO_2_ and NH_2_, and green-optimized experimental structures from CSD.

Electron density at the critical point of the central ring (ρ(r)), potential-energy density (V(r)), and kinetic-energy density (G(r)) for the electrons at the critical point of the central ring have been correlated with the α ring, and is presented in Figure 1. The substitution of both the central-aliphatic and side-aromatic ring affects the α angle. Therefore, it is interesting to determine the relationship between the electron density of the central-ring-critical point (RCP) and the α angle. The potential energy density (V(r)) is affected by the pressure exerted on the electrons at the RCP by other electrons. In contrast, kinetic energy density (G(r)) is related to the pressure exerted by the electrons in the RCP on other electrons [39]. The QTAIM parameters for the RCP located in the centre of the aliphatic ring are the most sensitive to the substituent in the central ring and strongly depend on the α angle. For most investigated structures, an increase in the α angle causes an increase in the electron density at the RCP for the aliphatic ring. The value of the potential energy density of electrons at the ring-critical point for the central ring decreases as the value of the α angle for the above-mentioned structures increases, while the kinetic energy density of electrons at the critical point of the central ring increases as the value of the α angle increases. Similar correlations were obtained in a previous work [33], where the effect of substituents on the α angle of phenothiazine, 9H-thioxanthene, and 9,10-dihydroanthracene derivatives was studied. No correlation was found for the QTAIM parameters of the RCP and the α angle for anthraquinones with a substituent in the side ring. This is most likely due to the bending of the substituted aromatic ring.

The correlations in Figure 1 show that the electron density as well as potential and kinetic-energy density at the RCP of the aliphatic ring are sensitive to the α angle. The planarity of the anthrone molecule is connected with the decreasing of the electron density and mobility of the electrons at the RCP when, for nonplanar compounds, the electron density and mobility of electrons are higher. The reverse tendency is observed for potential-energy density at the RCP. The general correlation of the QTAIM parameters can be split into categories corresponding to individual compounds. The most sensitive to the α angle are the QTAIM parameters for the aliphatic RCP for the anthrones substituted in the aliphatic ring when substitution of the side ring causes only limited changes of the electron density at the aliphatic RCP. Substitution of the anthraquinone side ring does not influence the electron density of the central ring.

### 2.3. Bond Ellipticity

Other fragments of the molecule sensitive to changes in flatness of the central aliphatic ring are the C–C bonds common for the aliphatic and aromatic ring. Participation in the aromatic ring is connected with the increasing of electron density compared with a typical aliphatic bond. Ellipticity of the electron density at the bond-critical point (BCP) gives information about the π-nature of the C–C bond. It is not possible to characterize the heteroatom—carbon bonding the measure of the π-nature—because of the free electron pair on the heteroatom [40], although ellipticity changes for the C=O bond are noticeable. The ellipticity and the length of the C–C bonds in the central ring are affected by substituents in both the middle and the side-aromatic ring. In order to better understand the change of ellipticity of the aliphatic ring under substitution, the ellipticity was compared with these for the unsubstituted structures.

Symmetrical substituents in the central ring cause the same changes in ellipticity and length in the bonds C8a–C10a and C4a–C9a, C8a–C9 and C9–C9a, and C10a–C10 and C10–C4a (atom numbering according to Scheme 1) relative to the unsubstituted molecule.

The highest ellipticity suggesting the π-nature of the bonds was observed for the bonds C8a–C10a and C4a–C9a, with lengths in the range of 1.38–1.44 Å. The C10a–C10 and C10–C4a bonds are characterized by the lowest ellipticity and the lengths within the range of 1.46–1.54 Å typical for single bonds.

Substitution of the amino groups in the aromatic side ring of anthrones causes an increase of ellipticity for most bonds. The exceptions are the C10a–C10 and C10–C4a bonds with a single amino substitution. This substitution has the greatest effect on the ellipticity of the bonds located closer to the substituted side ring (i.e., C10–C4a, C4a–C9a, and C9–C9a). A particularly large increase of ellipticity is observed for the C9–C9a bond.

Amino groups substituted in the anthraquinone side ring also cause an increase of the ellipticity for most bonds. Only for the C8a–C10a bond is the ellipticity very close to the ellipticity for the same bond in the unsubstituted molecule. However, a single substitution of the amino group in anthraquinones caused a slight decrease of ellipticity of the C10–C4a bond. As with anthrones, amino substitution has the greatest effect on the ellipticity of the bonds closer to the substituted side ring. The largest increase in ellipticity is observed for the C9–C9a bond.

Substitution of nitro groups in anthrones causes an increase of the ellipticity of the C8a–C9, C8a–C10a, C10a–C10, and C10–C4a bonds. In the case of the C4a–C9a bond, an increase of ellipticity occurs for one and disubstituted structures. On the other hand, substitution with nitro groups of the side ring causes a decrease of the ellipticity of the C9–C9a bond. The greatest changes in ellipticity relative to the unsubstituted molecule were observed for the C9–C9a bond.

The substitution of nitro groups in anthraquinones causes an increase of the ellipticity of the C8a–C9, C8a–C10a, C10a–C10, and C4a–C9a bonds. For the C10–C4a and C9–C9a bonds, ellipticity decreased. In this case, substitution also has the most significant effect on change of the C9–C9a bond ellipticity relative to the unsubstituted structure.

**Figure 2:**
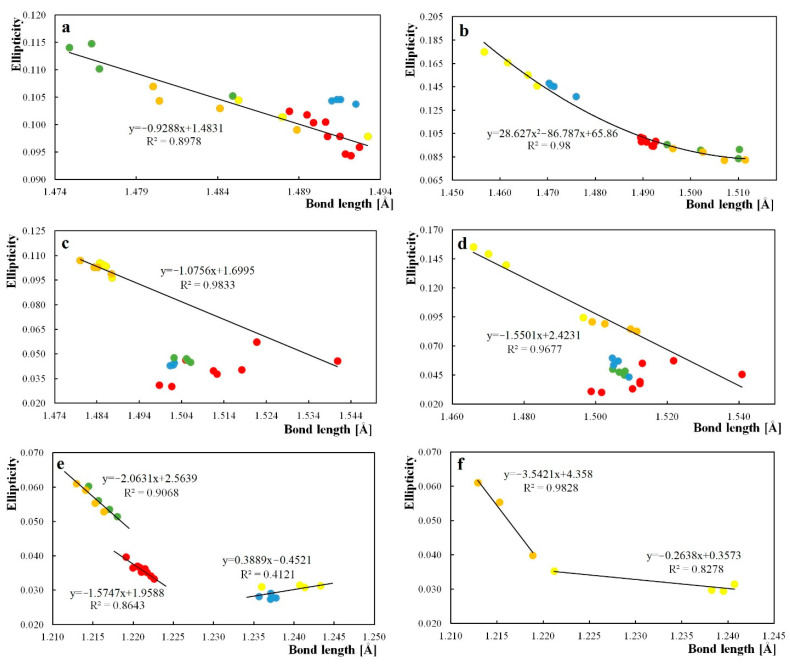
Correlations between ellipticity and bond length of the central aliphatic ring, (**a**)—C8a–C9 bond, (**b**)—C9–C9a bond, (**c**)—C10a–C10 bond, (**d**)—C10–C4a bond, (**e**)—C9=O bond, and (**f**)—C10=O bond, where red-anthrones with a substituent in the central ring, blue-anthrones with a substituent NH_2_, green-anthrones with a substituent NO_2_, yellow-anthraquinones with a substituent NH_2_, and orange-anthraquinones with a substituent NO_2_. The C8a–C9 bond for anthrones substituted with the NH_2_ group in the side ring was not included in the trend line.

Substitution with amino and nitro groups influences the geometry of anthrones and anthraquinones, so a correlation of ellipticity of electron density at BCP and bond lengths can be expected. Correlations of the bond length and ellipticity for the central-ring bonds with the correlation lines are presented in Figure 2. The best fit was obtained for the C9–C9a bond. For bonds C8a–C10a and C4a–C9a, no correlation was found. For the C9=O bond, three trend lines were drawn: for structures with substituents NH_2_ and NO_2_ and for structures with substituents in the middle ring. The same was done with regard to the C10=O bond. The C8a–C9 bond for anthrones substituted with the NH_2_ group in the side ring was not included in the trend line. A lack of oxygen at C10 carbon results in less electron-density flow between the side, substituted aromatic rings and the central-aliphatic ring in anthrone. Therefore, substitution of the anthrone lateral ring with electron-donor and electron-withdrawing substituents slightly affects the C10a–C10 and C10–C4a bonds. In addition, the different nature and size of substituents in the central ring has a significant impact on the ellipticity and length of the C10a–C10 and C10–C4a bonds. The trend line for the C10a–C10 and C10–C4a bonds was determined on the basis of anthraquinones with NH_2_ and NO_2_ substituents in the side-aromatic ring and two anthrone structures with a substituent in the central-aliphatic ring (NH_2_ and C(CH_3_)_3_). The changes of ellipticity confirm that substitution of the side ring of anthrones and anthraquinones does not significantly influence the bonds common for the aliphatic and aromatic ring.

### 2.4. *Harmonic* Oscillator Model of Aromaticity

In order to study the effect of substituents on ring aromaticity, the HOMA geometric index was proposed [22]. For the benzene aromatic ring, the HOMA index is equal to 1; for cyclohexane, it is zero; and for the antiaromatic ring, it is negative.

(1) $$HOMA=1-\alpha /n\sum_{i=1}^{n}{\left(R_{opt}-R_{ij}\right)}^{2}$$

*R_opt_*—the optimized CC bond length of a perfectly aromatic system and equals 1.388 Å
*R_ij_*—determined bond length
α—standardization constant of 257.7
*n*—number of bonds

**Figure 3:**
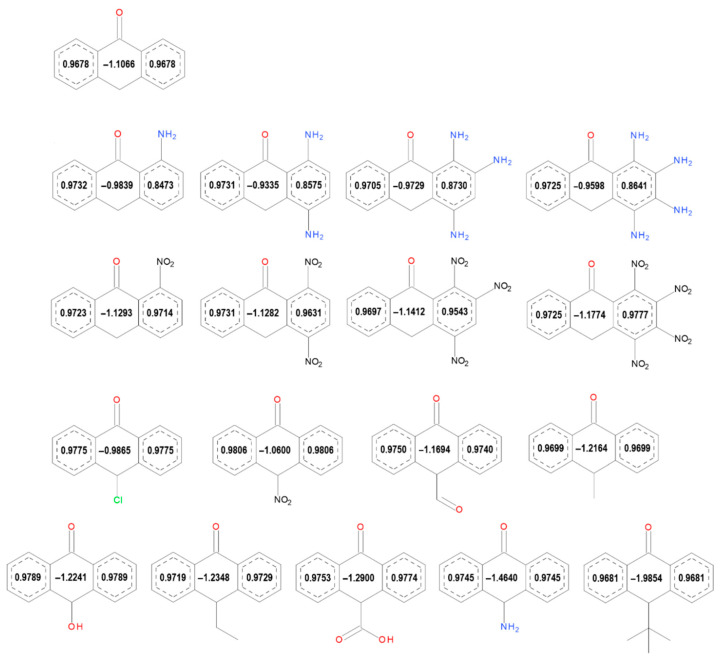
*Harmonic* oscillator model of aromaticity (HOMA) parameters for anthrone structures with a substituent in the side aromatic ring and in the central aliphatic ring.

**Figure 4:**
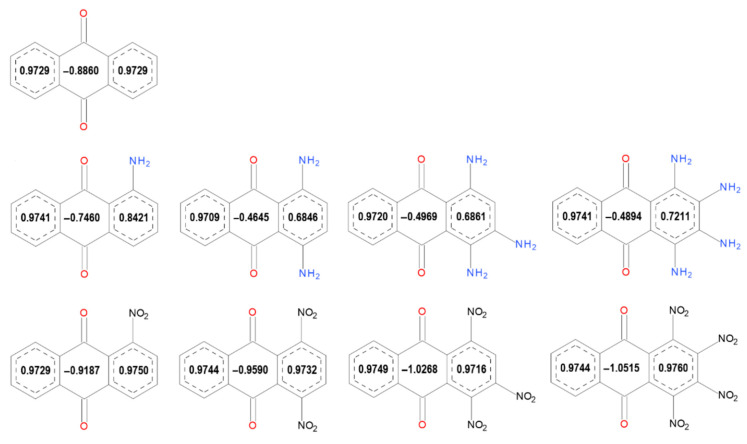
HOMA parameters for anthraquinone structures with a substituent in the side aromatic.

**Figure 5:**
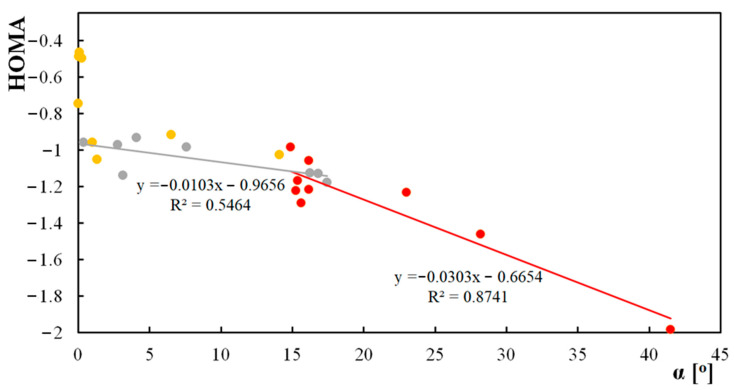
Correlations between HOMA parameters for the central ring and α angle: yellow-anthraquinones with a substituent NH_2_ and NO_2_, red-anthrones with a substituent in the central ring, and grey-anthrones with a substituent NH_2_ and NO_2_ in the side ring.

The HOMA parameters for the investigated compounds are shown in Figure 3 and Figure 4 and the correlation of HOMA with α angle is shown in Figure 5. Analogous calculations of the aromaticity of the anthrone rings confirmed the aliphatic character of the central ring [19].

Of particular interest is the influence of substituents in the lateral aromatic ring on the aromaticity, not only of the aliphatic ring, but also on the aromaticity of the second, unsubstituted aromatic ring. This phenomenon was observed for all investigated compounds. The lowest HOMA value in the central ring was observed for the anthrone molecule substituted with four nitro groups in the aromatic side ring. The largest increase of the HOMA index for the central ring of anthraquinone was observed in the case of substitution of the aromatic side ring with two amino groups in para position. Electron density at the RCP of the central ring is significantly affected by the presence of the C=O. This is due to the formation of weak hydrogen bonds (H⋯O = 1.85 Å) between the carbonyl oxygen and the hydrogen of the amine substituent closest to the middle ring. Greater changes of the HOMA value relative to the unsubstituted structure were observed for the structures with substituents in the side ring of anthraquinones than for anthrones.

Changes of the HOMA values under substitution show that aromaticity of the side rings is influenced by donor and acceptor properties of the substituents as well as the size of the substituent. Substitution of the middle ring with Cl, NO_2_, CH_3_, OH, NH_2_, and C(CH_3_)_3_ does not influence the electron-density distribution in the aromatic rings, and the HOMA value for both aromatic side rings is the same.

A correlation between the HOMA parameter and the α angle was determined (Figure 5). For both anthrones with a substituent in the middle ring and anthrones with NO_2_ and NH_2_ substituents in the central ring, the HOMA value decreases as the alpha angle increases. When the alpha angle is low, the molecule is flat and the electron density in the central ring increases. Because, in anthraquinones, multiple substitution of the aromatic ring with electron-donor and electron-withdrawing substituents causes the substituted ring to be bend, no correlation between the HOMA parameter and the α angle was found. The value of the HOMA parameter of the central aliphatic ring shifts towards aromaticity after substitution of the side ring with an electron-donating substituent, while substitution of the side ring with an electron-withdrawing group causes a shift of the HOMA parameter value towards anti-aromaticity.

The demonstrated effect of the substitution, and especially the substitution in the central ring of anthrones, on changes in aromaticity and the correlated changes in geometry expressed by the α angle, shows the flexibility of the central ring. Consequently, a substitution can significantly change the geometry of the molecule and its properties. One of the most important problems with anthrone is the balance between the ketone and hydroxyl forms. As this equilibrium is known to depend on many structural factors, changes in the aromaticity of the rings, and the environment of the molecule [19,41], it is expected that a change in the aromaticity of the rings will affect the keto-enol equilibrium. This is especially important for compounds with therapeutic properties; therefore, based on the known structure, theoretical studies of the keto-enol equilibrium for compounds used as drugs should be carried out.

## 3. Conclusions

The change of the angle between the anthrone aromatic rings is associated with the change in electron density at the RCP of the central ring. The value of the HOMA parameter of the central aliphatic ring shifts towards aromaticity after substitution of the side ring with an electron-donating substituent, while substitution of the side ring with an electron-withdrawing group causes a shift of the HOMA parameter value towards anti-aromaticity. Aromaticity of the anthrone rings is affected by the electron-donating and electron-withdrawing properties and the size of the substituent linked to the aromatic side ring as well as to the central aliphatic ring. Substituents in the anthrone aromatic ring affect the geometry and electronic structure of the central ring. Substitution in the central ring has the greatest impact on the structure of the entire molecule.

## Figures and Tables

**Table 1:** α angle defined according to Scheme 2 for the investigated compounds. Angles for the optimized compounds (upper) and the angles for the crystal structure (lower).

	Name	α [°]	Structure
1	10*H*-anthracen-9-one	0.023	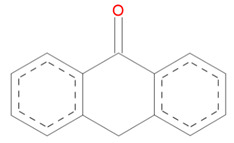
2	anthracene-9,10-dione	0.003	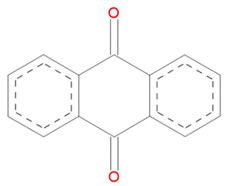
3	1-(3-chloro-4-methylphenyl)-3-(10-oxo-9,10-dihydroanthracen-9-yl)pyrrolidine-2,5-dione	18.346 14.252 [28]	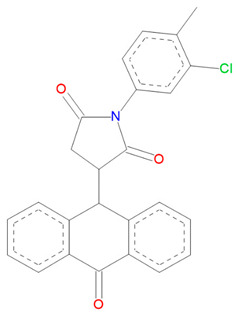
4	Methyl (*R*)-2-((4-fluorophenyl)(10-oxo-9,10-dihydroanthracen-9-yl)methyl)acrylate	18.715 25.727 [29]	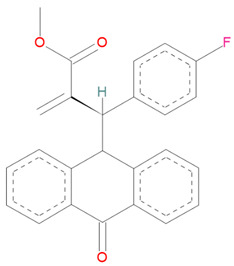
5	10-((1*S*,2*S*)-1-(4-Bromophenyl)-2-methyl-3-oxobutyl)anthracen-9(10*H*)-one	21.432 18.895 [29]	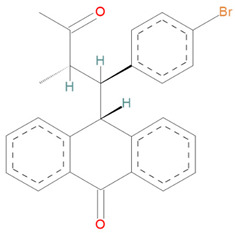
6	9-Oxo-10-diphenylphosphinoylanthracene	23.705 26.869 [30]	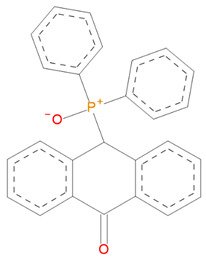
7	(*S*)-10-(1-(4-Chlorophenyl)-3-oxobutyl)anthracen-9(10*H*)-one	23.330 23.095 [31]	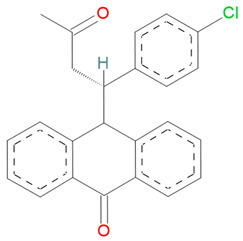
8	10-methyl-10*H*-anthracen-9-one	16.168 4.920 [27]	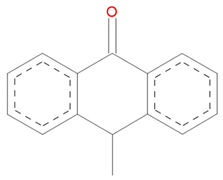
9	10-Isopropyl-9,10-dihydro-9-anthracenone	21.684 22.692 [27]	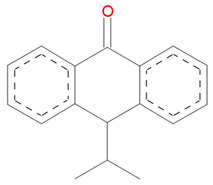
10	10-tert-butyl-10*H*-anthracen-9-one (axial position)	28.177 26.680 [27]	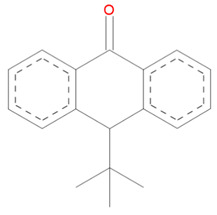
11	1,8-dihydroxy-10-(1-hydroxy-1,2-dihydronaphthalen-2-yl)anthracen-9(10*H*)-one	19.281 19.764 [32]	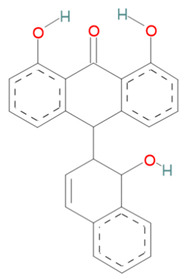
12	10-(6,7-dibromo-1-hydroxy-5,8-dimethyl-1,2-dihydronaphthalen-2-yl)anthracen-9(10*H*)-one	25.498 25.296 [32]	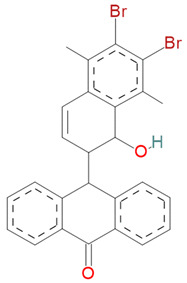
13	10-(6,7-difluoro-1-hydroxy-1,2-dihydronaphthalen-2-yl)-1,8-dihydroxyanthracen-9(10*H*)-one	19.547 18.505 [32]	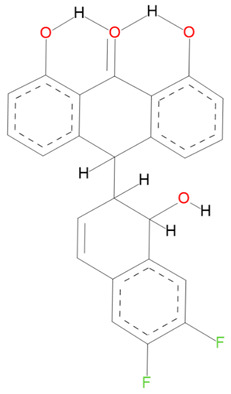
14	10-carbaldehyde-10*H*-anthracen-9-one	15.369	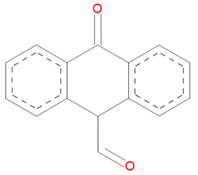
15	10-chloro-10*H*-anthracen-9-one	14.876	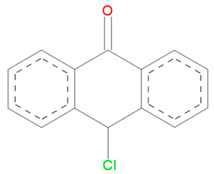
16	9-oxo-10*H*-anthracene-10-carboxylic acid	15.617	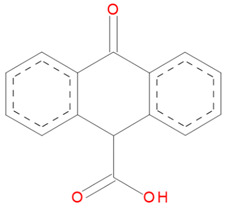
17	10-ethyl-10*H*-anthracen-9-one	22.978	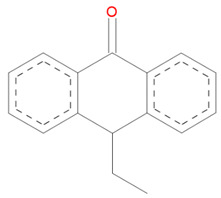
18	10-amino-10*H*-anthracen-9-one	28.192	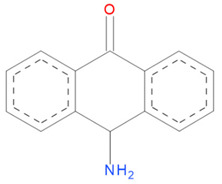
19	10-nitro-10*H*-anthracen-9-one	16.168	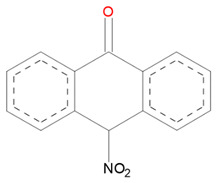
20	10-hydroxy-10*H*-anthracen-9-one	15.267	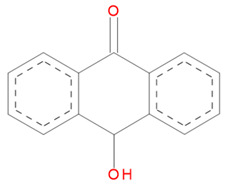
21	10-tert-butyl-10*H*-anthracen-9-one (equatorial position)	41.498	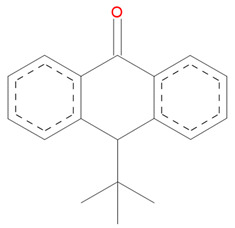
22	1-amino-10*H*-anthracen-9-one	7.569	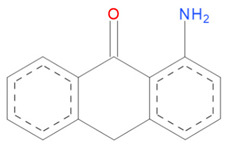
23	1,4-diamino-10*H*-anthracen-9-one	4.074	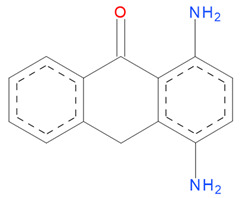
24	1,2,4-triamino-10*H*-anthracen-9-one	2.752	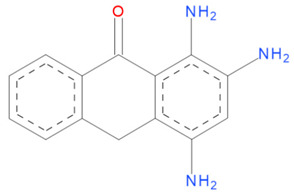
25	1,2,3,4-tetraamino-10*H*-anthracen-9-one	0.357	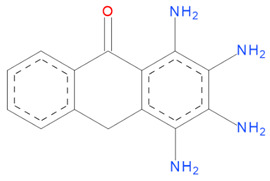
26	1-aminoanthracene-9,10-dione	0.008	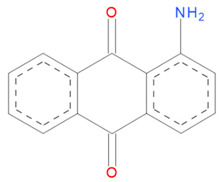
27	1,4-diaminoanthracene-9,10-dione	0.094	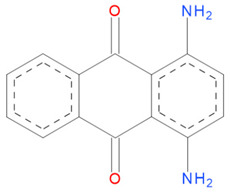
28	1,3,4-triaminoanthracene-9,10-dione	0.269	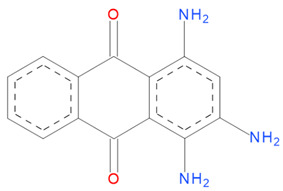
29	1,2,3,4-tetraaminoanthracene-9,10-dione	0.053	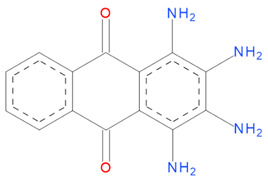
30	1-nitro-10*H*-anthracen-9-one	16.812	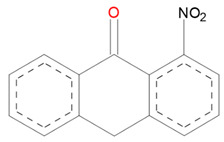
31	1,4-dinitro-10*H*-anthracen-9-one	16.22	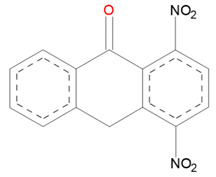
32	1,2,4-trinitro-10*H*-anthracen-9-one	3.137	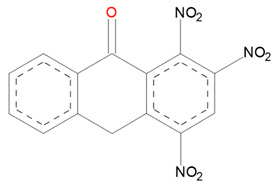
33	1,2,3,4-tetranitro-10*H*-anthracen-9-one	17.452	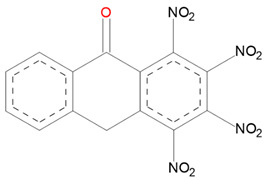
34	1-nitroanthracene-9,10-dione	6.528	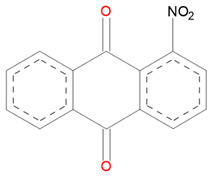
35	1,4-dinitroanthracene-9,10-dione	1.008	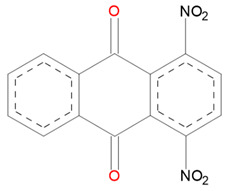
36	1,3,4-trinitroanthracene-9,10-dione	14.116	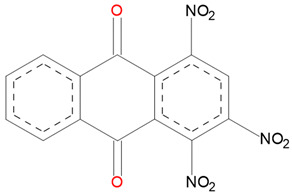
37	1,2,3,4-tetranitroanthracene-9,10-dione	1.305	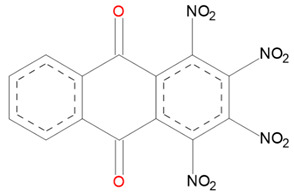

**Table 2:** β angle and C–N bond length for substituents in the aromatic side ring.

Molecule	The Number of the Carbon Atom to Which Substituent Is Attached	Single Substitution		Double Substitution		Triple Substitution		Quadruple Substitution	
		β Angle [°]	C–N Bond Length [Å]	β Angle [°]	C–N Bond Length [Å]	β Angle [°]	C–N Bond Length [Å]	β Angle [°]	C–N Bond Length [Å]
Anthrone with amino substituents	1	8.287	1.361	18.275	1.369	41.642	1.388	48.021	1.398
	2	—	—	—	—	48.465	1.410	52.29	1.415
	3	—	—	—	—	—	—	45.828	1.408
	4	—	—	43.760	1.415	42.485	1.412	46.847	1.414
Anthrone with nitro substituents	1	65.671	1.486	69.204	1.486	72.621	1.493	69.097	1.494
	2	—	—	—	—	33.269	1.485	53.625	1.488
	3	—	—	—	—	—	—	53.408	1.488
	4	—	—	34.192	1.482	38.135	1.486	58.199	1.486
Anthraquinone with amino substituents	1	0.588	1.358	11.006	1.362	8.797	1.361	33.212	1.370
	2	—	—	—	—	—	—	49.769	1.410
	3	—	—	—	—	43.815	1.394	50.137	1.402
	4	—	—	11.072	1.362	41.063	1.382	43.981	1.388
Anthraquinone with nitro substituents	1	70.053	1.486	70.416	1.487	73.663	1.488	67.81	1.494
	2	—	—	—	—	—	—	55.286	1.490
	3	—	—	—	—	34.506	1.487	55.309	1.489
	4	—	—	70.271	1.487	73.092	1.493	67.802	1.493

## Data Availability

The data can be obtained from the authors.

## Supplementary Materials

The following are available online, Table S1: Bond length and ellipticity for the central ring of the optimized compounds.

## Sample Availability

Samples of the molecular geometry are available from the authors.
