# Supplementary material for: Effect of Substitution of Hydrogen Atoms in the Molecules of Anthrone and Anthraquinone
Source: Molecules. 2021 Jan 19;26(2):502. doi: 10.3390/molecules26020502 (PMC7832899; doi:10.3390/molecules26020502)
Supplement: Supplementary file 1 [file molecules-26-00502-s001.pdf]

Table S1. Bond length and ellipticity for the central ring of the optimized compounds.

Compound	C8a-C9		C8a-C10a		C10a-C10		C4a-C10		C4a-C9a		C9a-C9	
	bond length [Å]	ε	bond length [Å]	ε	bond length [Å]	ε	bond length [Å]	ε	bond length [Å]	ε	bond length [A]	ε
10*H*-anthracen-9-one	1.4898	0.1027	1.4020	0.1981	1.5065	0.0450	1.5065	0.0450	1.4020	0.1981	1.4898	0.1027
anthracene-9,10-dione	1.4924	0.0955	1.4064	0.1808	1.4924	0.0955	1.4924	0.0955	1.4064	0.1808	1.4924	0.0955
10-methyl-10*H*-anthracen-9-one	1.4895	0.1018	1.4027	0.1954	1.5124	0.0379	1.5124	0.0379	1.4027	0.1954	1.4895	0.1018
10-formyl-10*H*-anthracen-9-one	1.4884	0.1025	1.4022	0.1990	1.5049	0.0465	1.5130	0.0553	1.4031	0.1967	1.4927	0.0986
10-chloro-10*H*-anthracen-9-one	1.4907	0.0979	1.4022	0.1992	1.4987	0.0310	1.4987	0.0310	1.4022	0.1992	1.4907	0.0979
9-oxo-10*H*-anthracene-10-carboxylic acid	1.4915	0.0979	1.4015	0.1994	1.5182	0.0405	1.5102	0.0334	1.4001	0.1990	1.4896	0.0983
10-ethyl-10*H*-anthracen-9-one	1.4906	0.1005	1.4046	0.1920	1.5115	0.0398	1.5124	0.0395	1.4031	0.1935	1.4900	0.1011
10-amino-10*H*-anthracen-9-one	1.4899	0.1004	1.4059	0.1912	1.5217	0.0574	1.5217	0.0574	1.4059	0.1912	1.4899	0.1004
10-nitro-10*H*-anthracen-9-one	1.4922	0.0944	1.4011	0.1987	1.5017	0.0303	1.5016	0.0303	1.4011	0.1987	1.4922	0.0943
10-hydroxy-10*H*-anthracen-9-one	1.4927	0.0959	1.4014	0.1919	1.5117	0.0052	1.5087	0.0138	1.4014	0.1974	1.4901	0.0992
10-tert-butyl-10*H*-anthracen-9-one	1.4918	0.0947	1.4113	0.1878	1.5408	0.0458	1.5408	0.0458	1.4113	0.1878	1.4918	0.0947
1-amino-10*H*-anthracen-9-one	1.4915	0.1046	1.3990	0.1982	1.5023	0.0447	1.5092	0.0435	1.4151	0.2056	1.4707	0.1462
1,4-diamino-10*H*-anthracen-9-one	1.4910	0.1044	1.3980	0.1989	1.5014	0.0430	1.5049	0.0538	1.4192	0.2149	1.4713	0.1456
1,2,4-triamino-10*H*-anthracen-9-one	1.4913	0.1046	1.3980	0.1986	1.5012	0.0431	1.5046	0.0599	1.4201	0.2159	1.4760	0.1369
1,2,3,4-tetraamino-10*H*-anthracen-9-one	1.4925	0.1038	1.3974	0.1993	1.5020	0.0435	1.5062	0.0573	1.4207	0.2148	1.4703	0.1484
1-aminoanthracene-9,10-dione	1.4932	0.0979	1.4035	0.1814	1.4875	0.0966	1.4965	0.0945	1.4206	0.1893	1.4678	0.1461
1,4-diaminoanthracene-9,10-dione	1.4853	0.1045	1.4021	0.1807	1.4853	0.1045	1.4659	0.1552	1.4417	0.1922	1.4659	0.1552
1,3,4-triaminoanthracene-9,10-dione	1.4879	0.1014	1.4016	0.1806	1.4846	0.1054	1.4750	0.1400	1.4415	0.1909	1.4567	0.1751
1,2,3,4-tetraaminoanthracene-9,10-dione	1.4880	0.1014	1.4013	0.1807	1.4861	0.1035	1.4701	0.1493	1.4398	0.1905	1.4616	0.1660
1-nitro-10*H*-anthracen-9-one	1.4849	0.1053	1.4014	0.1990	1.5061	0.0451	1.5080	0.0451	1.4031	0.2020	1.4950	0.0958
1,4-dinitro-10*H*-anthracen-9-one	1.4767	0.1102	1.3998	0.1989	1.5053	0.0465	1.5081	0.0486	1.4075	0.1996	1.5021	0.0911
1,2,4-trinitro-10*H*-anthracen-9-one	1.4762	0.1148	1.3980	0.2014	1.5022	0.0479	1.5047	0.0501	1.4092	0.1943	1.5102	0.0915
1,2,3,4-tetranitro-10*H*-anthracen-9-one	1.4749	0.1141	1.4009	0.2011	1.5051	0.0472	1.5065	0.0476	1.4034	0.1933	1.5100	0.0837
1-nitroanthracene-9,10-dione	1.4889	0.0991	1.4056	0.1822	1.4874	0.0989	1.4990	0.0911	1.4071	0.1856	1.4962	0.0924
1,4-dinitroanthracene-9,10-dione	1.4841	0.1030	1.4047	0.1830	1.4841	0.1030	1.5026	0.0893	1.4086	0.1905	1.5025	0.0893

1,3,4-trinitroanthracene-9,10-dione	1.4804	0.1044	1.4045	0.1840	1.4833	0.1029	1.5097	0.0848	1.4078	0.1869	1.5071	0.0823
1,2,3,4-tetranitroanthracene-9,10-dione	1.4800	0.1070	1.4052	0.1832	1.4800	0.1070	1.5114	0.0827	1.4046	0.1871	1.5114	0.0827
